# Differential regulation of radioadaptation by quercetin between human normal and cancer cells

**DOI:** 10.1016/j.ctro.2025.101099

**Published:** 2025-12-17

**Authors:** Chujie Li, Xiaojun Li, Rianne Biemans, Rui Zhang, Ming Zhang, Ludwig J. Dubois

**Affiliations:** a The M-Lab, Department of Precision Medicine, GROW – Research Institute for Oncology and Reproduction, Maastricht University, Maastricht, the Netherlands; b Zhongshan Hospital of Traditional Chinese Medicine, Zhongshan 528400, PR China; c Department of Radiotherapy, GROW – Research Institute for Oncology and Reproduction, Maastricht University, Maastricht, the Netherlands; d Hainan University-HSF/LWL Collaborative Innovation Laboratory, College of Food Sciences & Engineering, Hainan University, Haikou 570228, PR China

**Keywords:** Quercetin, Radiotherapy, Radioadaptation, Antioxidant, DNA damage

## Abstract

**Objective:**

Radiotherapy is a primary treatment for many cancers, but its efficacy is often limited by collateral damage to healthy tissues. Radioadaptation, a phenomenon where low-dose radiotherapy (LDRT) enhances a cell’s ability to withstand subsequent high-dose radiation, occurs in normal cells but is generally absent in cancer cells. Quercetin, a natural flavonoid with antioxidant and anticancer properties, has been proposed as a potential radiomodulator. This study aimed to investigate whether quercetin could differentially regulate the radioadaptive response in human normal breast epithelial versus breast cancer cells.

**Methods:**

Cell viability, clonogenic survival, oxidative stress, and DNA damage responses were assessed in MCF10A and MCF7 cells following treatment with LDRT (0.1 Gy), quercetin, and high-dose radiation. NQO1 and NRF2 expression levels were measured using RT-qPCR, Western blotting, and immunofluorescence. DNA damage was evaluated by γ-H2AX foci and p-ATM levels.

**Results:**

In MCF10A cells, LDRT pre-treatment enhanced resistance to subsequent radiation, which was further potentiated by quercetin, as shown by increased cell viability (p = 0.007), increased surviving fraction (enhancement ratio = 0.85, at 10 % surviving fraction), enhanced adaptation at 4 h on NQO1 mRNA (p < 0.01) and protein expression (p < 0.01), with a modest effect at 24 h on NQO1 mRNA (p = 0.890) and protein (p = 0.453) and reduced ROS level at 24 h (p = 0.021). Quercetin promoted NRF2 delocalization (p = 0.005). In contrast, MCF7 cells showed no radioadaptive response, and quercetin even increased radiosensitivity (enhancement ratio of surviving fraction = 1.12, at 10 surviving fraction) by maintaining ROS levels and DNA damage.

**Conclusion:**

Quercetin selectively enhances radioadaptation in normal cells by activating antioxidant pathways and reducing DNA damage, while preserving or amplifying radiosensitivity in cancer cells. These findings support quercetin may serve as a potential radiomodulating agent with favorable safety for increasing the therapeutic window of radiotherapy.

## Highlights

• Quercetin enhances low-dose radiation–induced adaptive response in normal MCF10A breast epithelial cells but not in MCF7 cancer cells.
• Quercetin promotes NRF2 nuclear translocation and NQO1 expression, thereby reducing ROS levels and residual DNA damage in radioadapted normal cells.
• In cancer cells, quercetin does not induce radioadaptation, but increases radiosensitivity instead, suggesting a selective radiomodulating effect that widens the therapeutic window.

## Introduction

Radiotherapy remains a cornerstone in the treatment of various malignancies, including breast cancer; however, its therapeutic success is often limited by damage to the surrounding healthy tissues [1]. One promising strategy to mitigate these effects is radioadaptation, a phenomenon where preconditioning with low-dose radiotherapy (LDRT) prepares cells to better withstand subsequent higher-dose irradiation [2], [3]. This effect is often absent in cancer cells due to their impaired stress response and genomic instability [4], [5], [6]. However, the molecular mechanisms underlying radioadaptation are not fully understood, and pharmacological agents that can enhance this protective effect without compromising radiotherapy’s anticancer efficacy are highly sought after.

Quercetin, a naturally occurring flavonoid found in fruits and vegetables, has attracted considerable attention due to its broad spectrum of biological activities, including antioxidant, anti-inflammatory, and anticancer properties [7]. Its potential role as a radiomodulator has been explored in recent years, with evidence suggesting that it may enhance cellular defense mechanisms in normal cells while exerting pro-oxidant and cytotoxic effects in cancer cells [8], [9]. Despite this dual functionality, the effects of quercetin on radioadaptation, as well as the underlying mechanisms, remain poorly understood.

In this study, we investigated whether quercetin could enhance the radioadaptive response in normal human breast epithelial cells while preserving its radiosensitizing effect in breast cancer cells. We assessed cell viability, clonogenic survival, oxidative stress levels, and DNA damage responses following combined treatment with LDRT priming, quercetin, and high-dose irradiation. Our findings reveal that quercetin selectively activates antioxidant pathways and attenuates DNA damage responses in normal radioadaptive cells, but not in cancer cells, supporting its potential application as a natural radioprotective agent in cancer therapy.

## Materials and methods

*Chemicals and Reagents*.

Quercetin·2H_2_O (>95 % purity) was purchased from Acros Organics (Geel, Belgium) and dissolved in ethanol to a stock concentration of 10 mM, which was subsequently diluted in culture medium to its final working concentration.

### Cell culture

The human breast cancer cell line MCF-7 (luminal A) was cultured in Dulbecco’s Modified Eagle Medium (DMEM, Sigma-Aldrich) supplemented with 10 % fetal bovine serum (FBS, Serana). The human breast epithelial cell line MCF-10A was cultured in DMEM/Ham’s F12 (1:1; Sigma-Aldrich, Gibco), supplemented with 10 % FBS, 10 μg/mL insulin (Roche), 20 ng/mL epidermal growth factor (R&D Systems), 0.5 μg/mL hydrocortisone (Sigma-Aldrich) and 100 ng/ml cholera toxin (Sigma-Aldrich). Cell lines were cultured in a humidified incubator at 37 °C with 5 % CO_2_ and were short tandem repeat-authenticated and confirmed to be mycoplasma-free by using the MycoAlert™ Mycoplasma Detection Kit (Lonza).

### Cell viability assay

Cells were seeded in 96-well plates (5000 cells per well), allowed to attach overnight and treated for 1 h with various concentrations of quercetin (3.125–100 µM), or DMEM as vehicle control. Cells were then irradiated (0.1 Gy) using a MultiRad225 (225 kV, 17.8 mA, 2.7 Gy/min, 0.3 mm Cu filtration, 15.4x15.4 cm FOV, SSD 38 cm; Precision X-Ray Irradiation Inc). 24 h later, cells were irradiated with 5 or 10 Gy and allowed to grow for another 24 h. Cell viability was determined using the MTT Cell Viability Reagent (Thermofisher). Briefly, the media was removed and MTT working solution (diluted in PBS, 1 mg/ml) was added to the wells, and incubated for one hour at 37 °C, protected from light. Absorbance (573 nm) was measured using a SpectraMax iD3 microplate reader (Molecular Devices). A non-toxic dose of quercetin was selected for further experiments.

### Clonogenic assay

Cells were seeded in 60 mm dishes (5x10^5^ cells/dish), allowed to attach overnight and treated with quercetin (12.5 µM) for 1 h. Cells were then irradiated (0.1 Gy) using a MultiRad225 (225 kV, 17.8 mA, 2.7 Gy/min, 0.3 mm Cu filtration, 15.4x15.4 cm FOV, SSD 38 cm; Precision X-Ray Irradiation Inc.). 24 h later, cells were irradiated again to produce adaptative cells with doses ranging from 2.5 to 10 Gy. Immediately after irradiation, cells were trypsinized and seeded as single cells in 60 mm dishes in triplicate, at optimized cellular density for clonogenic survival assay. Colonies were allowed to grow for 8–16 days, depending on each cell line. Cells were fixed and stained with 70 % ethanol, 0.4 % methylene blue, and colonies containing more than 50 cells were counted manually. Surviving fractions were calculated based on plating efficiency and were fit to a linear quadratic model as a function of dose, utilizing weighted least squares regression. The parameters of the clonogenic cell survival curves (A, B) were compared, and significance was tested using a log-rank curve comparison [10].

### DCFH-DA staining

After treatment, cells were stained with DCFH-DA (10 µ M) at 37°C for 40 min. The fluorescence was measured using a SpectraMax iD3 microplate reader (Molecular Devices) at excitation/emission wavelengths of 485/538 nm.

### Quantitative polymerase chain reaction (qPCR)

Total RNA was isolated from cells using the NucleoSpin RNA kit (Macherey Nagel) according to the manufactur’s protocol. mRNA was reverse transcribed using the iScript cDNA synthesis kit (Biorad) followed by SYBR-green based quantitative reverse transcription PCR (RT-qPCR) using the SensiMix SYBR high-ROX kit (GC Biotech). mRNA expression was analyzed using the primers described in Table 1.

**Table 1:** Quantitative real-time PCR primer sequences.

Target genes	Forward (5′-3′)	Reverse (5′-3′)
*NQO1*	GAAGAGCACTGATCGTACTGGC	GGATACTGAAAGTTCGCAGGG
*β-actin*	CATGTACGTTGCTATCCAGGC	CTCCTTAATGTCACGCACGAT

### Immunofluorescence

For NRF2 staining, cells were fixed 24 h after the final irradiation with 4 % paraformaldehyde (PFA) for 15 min on ice, followed by permeabilization with 0.5 % Tween-20 in PBS for 20 min at room temperature (RT). To reduce non-specific antibody binding, cells were then incubated with 5 % normal goat serum and 0.05 % Tween-20 in PBS for 30 min at RT. Cells were incubated with rabbit anti-human NRF2 primary antibody (1:200; Proteintech, 16396–1-AP), and detection was performed using goat anti-rabbit Alexa Fluor 488 (1:500, Invitrogen, 11001). Nuclei were counterstained with DAPI (1:500, Merck, D9542).

For γH2AX staining, cells were fixed 24 h after the final irradiation in ice-cold methanol for 15 min on ice, followed by permeabilization with 0.2 % Tween-20 in PBS for 20 min at RT. Non-specific antibody binding was blocking using 5 % normal goat serum and 0.02 % Tween-20 in PBS for 30 min at RT. Cells were incubated with a rabbit anti-human γH2AX primary antibody (1:500, Merck, JBW301), followed by detection using goat anti-rabbit Alexa Fluor 488 (1:500, Invitrogen, 11001). Nuclei were counterstained with DAPI (1:500, Merck, D9542).

All samples were imaged using a Leica SPE confocal microscope, and images were processed using Fiji (ImageJ). For NRF2, the nuclear-to-cytoplasmic (N/C) ratio of fluorescence intensity was calculated to assess nuclear translocation. Nuclear regions were defined using DAPI staining, and cytoplasmic regions were manually selected by excluding the DAPI-positive area. Mean fluorescence intensities in both compartments were measured, and the ratio was calculated for each cell. For γH2AX, DNA damage was quantified by counting γH2AX foci per nucleus. Nuclear masks were generated based on DAPI staining, and foci were identified within each nucleus after thresholding.

### Immunoblotting

Cells were lysed in RIPA buffer (50 mM HEPES-KOH, pH 7.5, 150 mM KCl, 1 Mm EDTA, 2 mM β-mercaptoethanol, 0.2 % Tween-20 (Sigma Aldrich, R0278) with protease inhibitor (Roche, 4693124001) and sonicated 3 × 5 s at 10 MHz. Samples (30 µg) in Laemmli sample buffer were loaded on a polyacrylamide gel, separated by SDS-PAGE, transferred to PVDF membranes (VWR, 10600023) using a wet transfer system. Membranes were blocked in 5 % non-fat milk in TBST for 1 h at room temperature and incubated overnight at 4 °C with primary antibodies against NQO1 (1:2000; Cell Signaling Technology, 62262), p-ATM (1:2000; Cell Signaling Technology, 5883). α-Tubulin (1:1000, Sigma Aldrich, T7451) or Vinculin (1:1000, Sigma Aldrich, V9131) were used as loading controls. Primary antibodies were visualized using HRP-link secondary antibodies (anti-rabbit, anti-mouse, 1:5000; Cell Signaling Technology, 7076S, 7074S). Super Signal West Pico chemiluminescent substrate (Thermo Scientific, 34579) was used for detection [11], [12].

### Statistical analysis

GraphPad Prism software (version 9.0) was used to perform statistical analyses. All results are expressed as mean ± SEM. The results were evaluated using one-way ANOVA with Tukey’s multiple comparison test or Student's *t*-test when appropriate. Values of p < 0.05 were considered statistically significant.

## Results

### Quercetin enhances radioadaptation in normal cells without compromising its anticancer effects

**Fig. 1:**
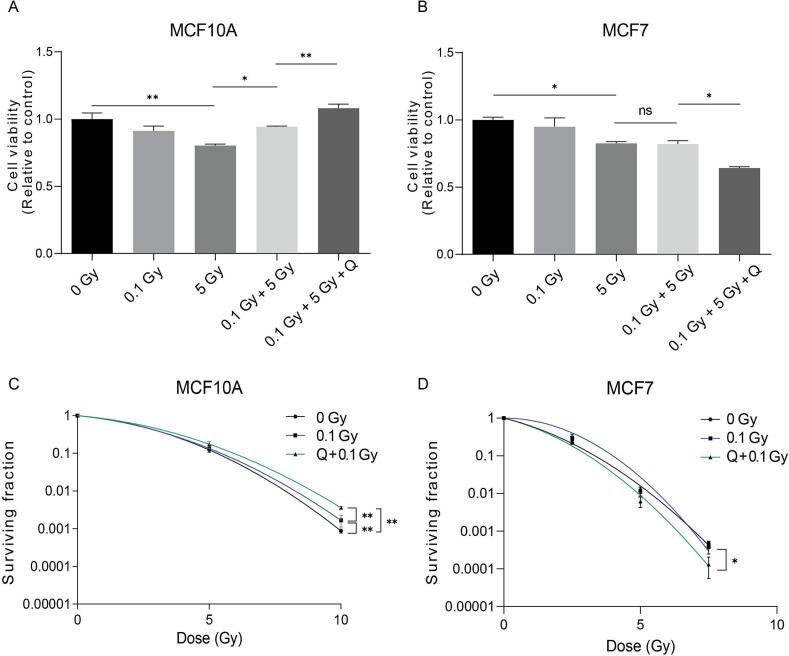
Quercetin enhances radioadaptation in normal MCF10A cells but sensitizes cancer MCF7 cells to radiation. (A–B) Cell viability in MCF10A (A) and MCF7 (B) cells following treatment with quercetin (12.5 μM), low-dose radiation (0.1 Gy) and subsequent high-dose radiation (5 Gy). (C–D) Surviving fractions determined by clonogenic assay for MCF10A (C) and MCF7 (D) cells under the same treatment conditions. Data is shown as mean ± SEM. * p < 0.05, ** p < 0.01.

In the current study, X-ray irradiation at 5 Gy significantly (p < 0.01) decreased the cell viability in MCF10A cells compared to the non-irradiated control group. This reduction in cell viability was significantly prevented (p < 0.034) by pre-irradiation with a 0.1 Gy priming dose. Moreover, 12.5 µM quercetin, selected as non-toxic concentration (supplementary Fig. 1) further amplified (p = 0.007) this radioadaptive effect in MCF10A cells (Fig. 1A). Parallel experiments in MCF7 cells revealed that 5 Gy irradiation decreased cell viability to a similar extent (p = 0.022), but the 0.1 Gy priming dose failed to modulate this effect (p = 0.999). Intriguingly, quercetin significantly (p = 0.031) inhibited cell viability in MCF7 cells compared to 0.1 + 5 Gy group (Fig. 1B). Colony formation assays confirmed that quercetin has radioadaptive effects (p < 0.01) in normal MCF10A cells (Fig. 1C), while exerting radiosensitizing effects (p = 0.044) in cancerous MCF7 cells (Fig. 1D).

### Quercetin reduces ROS accumulation and upregulates NQO1 expression in radioadapted MCF10A cells

**Fig. 2:**
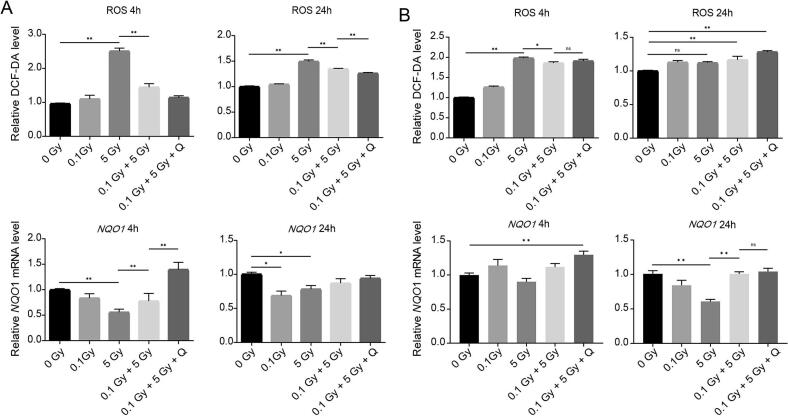
Effects of quercetin on ROS levels and NQO1 mRNA expression in radioadapted MCF10A and MCF7 cells. (A–B) ROS production and NQO1 mRNA expression were measured at 4 h and 24 h after 5 Gy irradiation in MCF10A (A) and MCF7 (B) cells, pre-treated or not with 0.1 Gy and quercetin. Data is shown as mean ± SEM. * p < 0.05, ** p < 0.01.

**Fig. 3:**
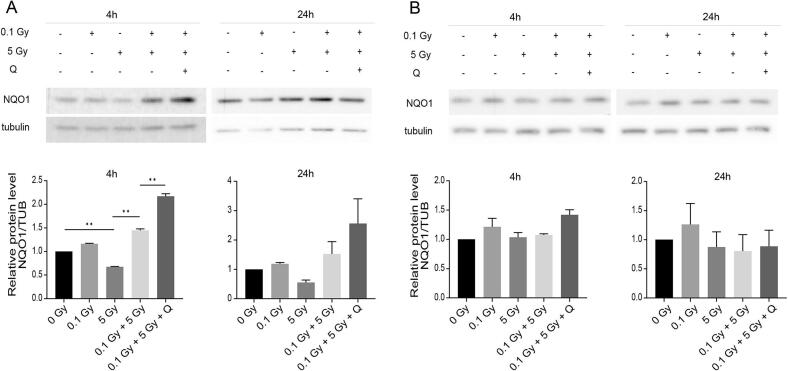
Quercetin upregulates NQO1 protein expression in normal, but not cancer cells. (A–B) NQO1 protein expression levels in MCF10A (A) and MCF7 (B) cells 4 h and 24 h after high-dose irradiation with or without prior LDRT and quercetin treatment. Data is shown as mean ± SEM. * p < 0.05, ** p < 0.01.

To investigate whether quercetin’s radioadaptive mechanisms involve antioxidant pathways, we analyzed NQO1 expression and ROS levels. In MCF10A cells, 5 Gy irradiation significantly suppressed NQO1 mRNA at 4 h (p < 0.01) and 24 h (p = 0.038) and protein expression at 4 h (p < 0.01) but not at 24 h (p = 0.938) and increased ROS levels at both 4 h (p < 0.01) and 24 h (p < 0.01) compared to controls. Pre-treatment with 0.1 Gy restored NQO1 mRNA expression at 4 h (p < 0.01) but not at 24 h (p = 0.509) and decreased ROS levels at 4 h (p < 0.01) and 24 h (p < 0.01). Quercetin significantly enhanced this adaptation at 4 h on NQO1 mRNA (p < 0.01) and protein expression (p < 0.01), with a modest effect at 24 h on NQO1 mRNA (p = 0.890) and protein (p = 0.453) (Fig. 2, Fig. 3). Quercetin significantly enhanced this adaptation at 24 h (p = 0.021) for the ROS levels. In contrast, MCF7 cells exhibited elevated ROS levels (p < 0.01) after 5 Gy irradiation, which remained unaltered by either 0.1 Gy priming dose or quercetin. NQO1 protein expression showed no significant changes across treatment groups (Fig. 2, Fig. 3).

### Quercetin activates the NRF2/NQO1 pathway in radioadapted MCF10A cells

**Fig. 4:**
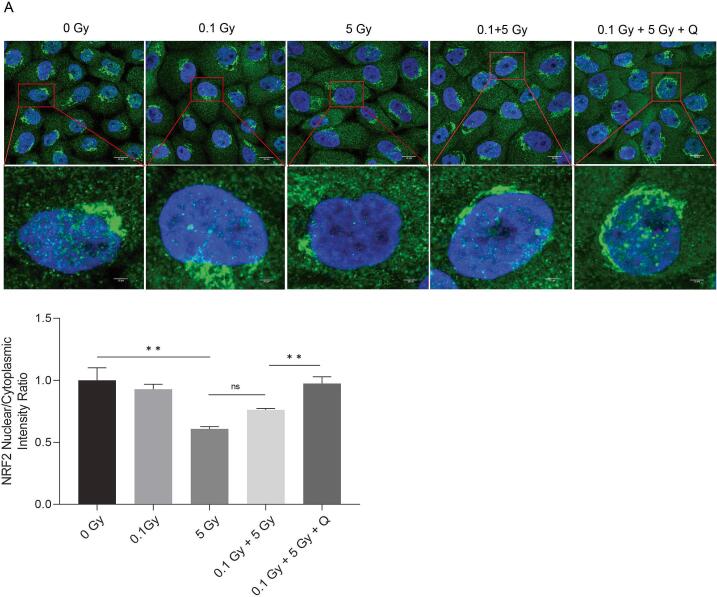
Quercetin restores NRF2 nuclear translocation in radioadapted MCF10A cells. Representative immunofluorescence images of NRF2 (green) and DAPI (blue) and quantification of the nuclear-to-cytoplasmic NRF2 fluorescence ratio in MCF10A cells 24 h after 5 Gy irradiation with or without prior LDRT and quercetin treatment (Scale bar: 10 μm). Data is shown as mean ± SEM. * p < 0.05, ** p < 0.01.

Given NRF2′s important role in antioxidant responses [13], we examined its protein expression 24 h after high-dose irradiation. To evaluate NRF2 activation, the nuclear-to-cytoplasmic NRF2 expression level ratio was measured. An increased nuclear-to-cytoplasmic ratio indicates translocation of NRF2 to the nucleus, reflecting its activation under oxidative stress conditions [14]. MCF10A cells exposed to 5 Gy showed reduced NRF2 nuclear translocation (p = 0.003) versus controls. The NRF2 nuclear-to-cytoplasmic ratio was slightly, although not significantly, elevated by 0.1 Gy priming dose (p = 0.35), and further significantly increased by quercetin in the 0.1 Gy + 5 Gy group (p = 0.005), similar to the ratio of the control group (Fig. 4).

### Quercetin attenuates ATM-dependent DNA damage response in radioadapted MCF10A cells

**Fig. 5:**
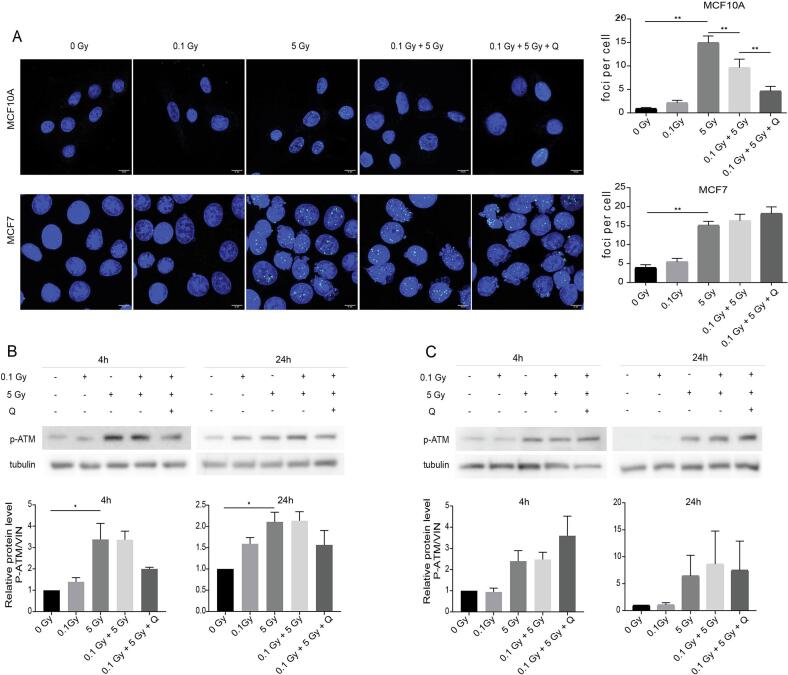
Quercetin attenuates DNA damage and ATM activation in normal but not cancer cells. (A) Immunofluorescence staining and quantification of γ-H2AX foci in MCF10A and MCF7 cells 24 h after high-dose irradiation with or without prior LDRT and quercetin treatment (Scale bar: 10 μm). (B–C) Representative western blots and quantification of phosphorylated ATM (p-ATM) levels in MCF10A (B) and MCF7 (C) cells at 4 h and 24 h after high-dose irradiation with or without prior LDRT and quercetin treatment. Data is shown as mean ± SEM. * p < 0.05, ** p < 0.01.

Residual DNA damage was assessed using the ionizing radiation-induced DNA damage marker (γ-H2AX) 24 h after irradiation [15], which was increased (p < 0.01) in MCF10A cells upon 5 Gy without priming, compared to the control non-irradiated group. Compared to 5 Gy group, pretreatment priming with 0.1 Gy significantly (p = 0.001) attenuated this increase. Quercetin treatment further decreased (p = 0.009) residual DNA damage. MCF7 cells exhibited a similar 5 Gy-induced DNA damage (p < 0.01), but neither 0.1 Gy pre-irradiation (p = 0.952) nor quercetin (p = 0.0864) altered the residual damage levels (Fig. 5A).

Activation of the DNA repair mechanisms was investigated by assessment of the expression levels of p-ATM [16]. A remarkable increase was observed following irradiation at 5 Gy, for MCF10A (p = 0.029), but not for MCF7 (p = 0.0869) cells, compared to non-irradiated group. Priming with 0.1 Gy did not affect p-ATM expression levels. Upon quercetin treatment, a tendency of p-ATM expression towards a decrease for MCF10A cells (p = 0.392), while an increase for MCF7 cells (p = 0.999) was observed, albeit not significant (Fig. 5B and C).

## Discussion

The present study demonstrates that quercetin enhances the radioadaptive response in normal human breast epithelial MCF10A cells while maintaining or even increasing radiosensitization in breast cancer MCF7 cells. Radiation adaptive response (RAR) refers to a phenomenon in which exposure to a low “priming” radiation dose induces increased resistance or reduced biological damage upon subsequent exposure to a higher “challenge” dose [3], [17]. This concept was first revealed in human lymphocytes, where a small priming dose of X-rays reduced chromosomal aberrations after a later high dose compared with cells receiving the high dose alone [3]. Although the classical RAR paradigm relies generally on a two-dose experiments, evidence now shows that adaptive responses can also be triggered by continuous low dose-rate exposure [18], [19], fractionated irradiation [20], and even cross-adaption between distinct radiation qualities such as γ-rays and particle irradiation [21], [22]. Bystander-mediated adaptive responses have also been demonstrated, in which non-irradiated cells acquire protection through intercellular signaling [23]. These findings suggest that RAR represents a much broader biological phenomenon. Studies showed that RAR characterizes by activation of antioxidant and DNA repair mechanisms at low doses (<0.2 Gy) [24], [25]. A consistent theme emerging from the literature is that normal cells frequently exhibit a robust RAR, whereas many tumor cells either fail to show RAR, show a blunted response, or even become more radiosensitive [26]. Several studies have showed that 75 mGy of X-ray irradiation fails to induce RAR in e.g. colon cancer cell [27], human gastric SGC7901 cells [28], or human leukemia cells [29], while normal cells exhibit RAR, characterized by enhanced DNA repair capacity, immune system and promoted proliferation [29], [30], [31], effects that disappear in some cancer cell types [6]. On the other hand, some studies report that certain tumor cells can exhibit RAR under specific conditions. For example, Wang et al. showed that 50–200 mGy X-rays induced RAR in A549 lung cancer cells prior to a 20 Gy challenge dose, although they attributed discrepancies across studies to differences in the chosen challenge dose [29], [32]. Similar contradictions are found in other models: low-dose priming (0.05 Gy) increased survival by 12.6 % in lung cancer H460 cells before 2 Gy [33], whereas prostate cancer (DU-145) cells showed no significant change under comparable conditions [34], suggesting that cell-type variability and genetic background strongly influence RAR outcomes. In this study, using the priming + challenging dose paradigm, we reveal a clear dichotomy in the cellular response to quercetin and low-dose radiotherapy (LDRT, 0.1 Gy) between non-tumorigenic and tumorigenic breast cell lines, highlighting quercetin's potential as a radiomodulator in cancer therapy.

A key observation of this study consistent with the above predictive frameworks is that LDRT (0.1 Gy) elicits a protective adaptive response in MCF10A cells, as shown by improved cell viability, enhanced clonogenic survival, reduced oxidative stress (ROS levels), and decreased DNA damage (γ-H2AX foci) after subsequent high-dose radiation (5 Gy). In contrast, this protective effect was not observed in MCF7 cells, where low-dose pre-irradiation did not significantly alter most responses, though ROS level decreased at 4 h, to 5 Gy. This is consistent with prior studies suggesting that radioadaptation is absent in some cancer cells [6], [35], [36], [37], [38]. Jiang et al. have observed a lack of adaptive response induced by LDRT (75 mGy) in U251, NCI-H446, K562 and HL60 cancer cell lines, and demonstrated that an adaptive response could be induced by pretreatment with LDRT in MRC-5 normal cells [6]. Another study also showed that LDRT (0.1 Gy) pretreatment significantly decreased 4 Gy induced cell death as an adaptive response prominent in normal cells (PK cells), and much less in tumor cells (ras-PK and 308 cells) [39]. Notably, quercetin potentiated these radioadaptive effects in MCF10A cells, not in MCF7 cells.

DNA damage response signaling, involving critical kinases like ataxia-telangiectasia mutated (ATM), and antioxidant response signaling (NRF2 pathway) have been suggested as possible mechanisms responsible for the difference in the induction of radioadaptive response by LDRT and quercetin between normal and tumor cells. Compared to healthy cells, cancer cells are characterized by increased formation of ROS. To avoid ROS-induced cell death, cancer cells restore their ROS homeostasis by increasing their antioxidant capacity [40]. NRF2 is a transcription factor, and activation of the NRF2-signaling pathway is an adaptive response to environmental and endogenous stresses. This response rendered animals resistant to chemicals and other forms of toxicity by inducing a variety of detoxification or antioxidant enzymes, such as NQO1 and HO1 [41]. In this study, NQO1 mRNA and protein expression was significantly increased in MCF10A cells when treated with 0.1 Gy and 5 Gy at 4 h, with a moderate increase maintained at 24 h. This suggests that the NQO1 antioxidant defense is promptly and effectively activated in normal breast epithelial cells in response to oxidative stress. In contrast, in MCF7 breast cancer cells, NQO1 mRNA expression increased only at 24 h, while protein levels remained unchanged. This delayed and incomplete response implies a dysregulation of post-transcriptional or translational control of antioxidant genes in cancer cells, reflecting an impaired or altered redox homeostasis compared to normal cells. NRF2 was detected in MCF10A cells and was upregulated, though not significantly, after low-dose pre-irradiation, indicating activation of NRF2-mediated antioxidant pathway. Quercetin further significantly increased nuclear accumulation of NRF2 in MCF10A cells, also leading to upregulation of antioxidant genes NQO1, reduced ROS levels, and enhanced resistance to radiation-induced oxidative damage. This pathway was largely inactive in MCF7 cells, indicating that the antioxidant-promoting effects of quercetin depend on an intact NRF2 signaling axis, which may be disrupted or overridden in cancerous cells [42], [43].

When ionizing radiation damages DNA, the kinase ATM phosphorylates H2AX around the DSB site [44]. The formed γH2AX foci recruit DNA repair proteins and signal activation of the DNA damage response. γH2AX serves as a quantitative biomarker for measuring the extent of DNA damage caused by radiotherapy [45]. ATM is a serine-threonine kinase of the phosphatidylinositol kinase-related kinase family that acts as an initial DNA damage sensor [46]. In MCF10A cells, low dose radiation pretreatment decreased γ-H2AX foci significantly, but not p-ATM protein expression. p-ATM is an early, transient, and localized signal at DSBs, while γH2AX is a signal amplifier downstream of ATM. This makes γH2AX more sensitive to radiation-induced changes than p-ATM [47]. Furthermore, γH2AX intensity does not always correlate linearly with p-ATM level [48]. Quercetin further significantly reduced γ-H2AX foci formation and slightly decreased p-ATM expression. In MCF7 cells, γ-H2AX foci formation and p-ATM protein expression did not significantly change between treatment groups. The observed differences in DNA damage markers between normal and tumor cells is in agreement with another study demonstrating that low dose radiation has distinct biological effects on normal and cancer cells [49], possibly due to hormesis [50], [51]. Consistent with quercetin’s antioxidant activity, quercetin further attenuated residual DNA damage in MCF10A cells. These changes suggest enhanced repair efficiency or lower burden of double-strand breaks of quercetin, possible via ATM-dependent pathways are effective radiation biomarkers in assessing DNA damage [52].

**Fig. 6:**
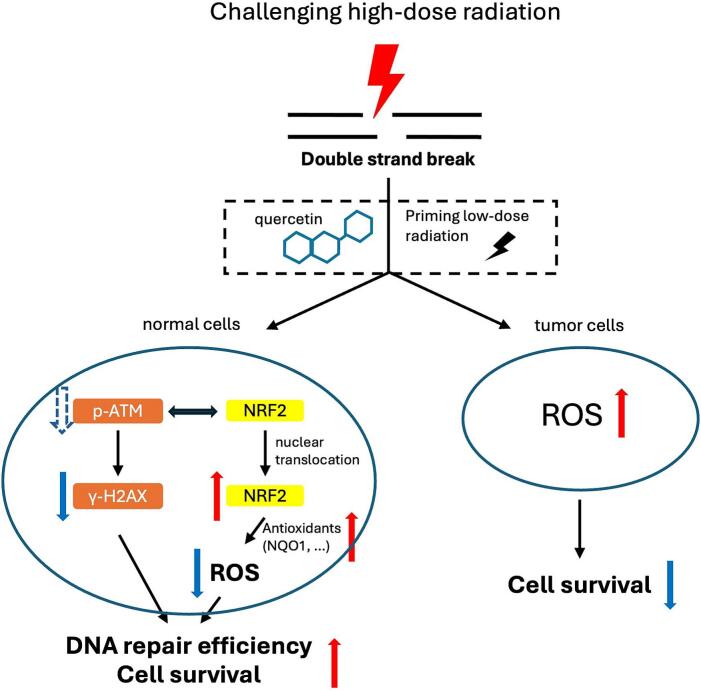
Schematic illustration of the proposed mechanism.

Low dose radiation can induce low levels of DSBs or ROS, resulting in the activation of ATM, which stimulates cell proliferation [53]. Previous study have demonstrated that ATM-dependent activation of NRF2 signaling and increased antioxidant expression were identified in LDR-induced anti-oxidant response, which was supported by the fact that blocking ATM with caffeine or ATM siRNA inhibited NRF2 nuclear accumulation [53]. In the present study, although p-ATM protein expression was only marginally affected in low dose pretreated and quercetin treated cells, the marked reduction in γH2AX foci formation and enhanced NRF2 delocalization also reflects coordinated regulation of DNA repair and redox homeostasis under low-dose conditions and quercetin treatment. The proposal mechanism suggests that LDRT and quercetin enhance antioxidant signaling while reducing DNA damage, and the interaction between ATM and NRF2 may also promote DNA damage repair, as shown in Fig. 6.

Importantly, while LDRT alone had minimal impact on MCF7 survival, the addition of quercetin markedly increased radiosensitivity, in agreement with previous reports of quercetin acting as a pro-oxidant in malignant cells [54], [55], [56]. This dual action-protection of normal cells and sensitization of cancer cells is the therapeutic goal.

While our findings support the use of quercetin as a radiation modulator, several limitations should be acknowledged. First, this study was conducted *in vitro*, and the tumor microenvironment *in vivo* may influence quercetin’s effects. Although the present study provides the promising protective effects of quercetin, its clinical translation remains challenging. One major limitation lies in the low bioavailability and poor absorption of quercetin *in vivo* [57], which significantly may restrict its therapeutic potential. Secondly, only one normal and one cancer cell line were examined, and future studies should validate these findings across a broader panel of matched cell types. Thirdly, using only one method for some indicators (e.g. ROS assessment) may introduce potential bias, although this approach is widely accepted and sensitive. In future studies, other complementary methods (e.g., chemiluminescence) could be employed to further validate and strengthen the findings. Additionally, in this study, we proposed that activation of the NRF2/NQO1 pathway plays a key role in the cellular defense against oxidative stress in normal cells, whereas this response is impaired in cancer cells. However, this mechanistic explanation remains relatively simplified. Other antioxidant and DNA repair pathways, such as SOD, CAT, PARP, and BRCA, may also contribute to the observed effects [58], [59], [60]. In addition, although changes in p-ATM were detected, they were only superficially characterized in the current study. Further investigations focusing on the functional efficiency of DNA repair and the involvement of specific signaling pathways are warranted to provide more comprehensive mechanistic evidence.

## Conclusion

Our findings add to the body of evidence that quercetin enhances the radioadaptive response in normal breast epithelial cells, while sensitizing breast cancer cells to radiation-induced damage. It appears that this is achieved by selective modulation of antioxidant defenses and DNA damage signaling. Future preclinical and clinical studies are warranted to fully unveil quercetin’s full potential in oncologic settings.

## CRediT authorship contribution statement

**Chujie Li:** Conceptualization, Methodology, Software, Validation, Formal analysis, Writing – original draft, Writing – review & editing. **Xiaojun Li:** Conceptualization, Validation, Writing – original draft. **Rianne Biemans:** Methodology. **Rui Zhang:** Methodology. **Ming Zhang:** Writing – review & editing, Supervision. **Ludwig J. Dubois:** Validation, Supervision.

## Declaration of competing interest

The authors declare the following financial interests/personal relationships which may be considered as potential competing interests: LJD has, outside of the submitted work, shares in the companies Convert Pharmaceuticals and LivingMed Biotech, and he is co-inventor of a non-issued patent on LSRT (N2024889).

## References

[1] Chen H.H.W., Kuo M.T. (2017). Improving radiotherapy in cancer treatment: promises and challenges. Oncotarget.

[2] Guéguen Y., Bontemps A., Ebrahimian T.G. (2019). Adaptive responses to low doses of radiation or chemicals: their cellular and molecular mechanisms. Cell Mol Life Sci.

[3] Olivieri G., Bodycote J., Wolff S. (1984). Adaptive response of human lymphocytes to low concentrations of radioactive thymidine. Science.

[4] Bonora M. (2021). Mitochondrial control of genomic instability in cancer. Cancers (Basel).

[5] Luo J., Solimini N.L., Elledge S.J. (2009). Principles of cancer therapy: oncogene and non-oncogene addiction. Cell.

[6] Jiang H. (2008). Low-dose radiation induces adaptive response in normal cells, but not in tumor cells: in vitro and in vivo studies. J Radiat Res.

[7] Kandemir K. (2022). Recent advances on the improvement of quercetin bioavailability. Trends Food Sci Technol.

[8] Patil S.L., Somashekarappa H., Rajashekhar K. (2012). Radiomodulatory role of Rutin and Quercetin in Swiss Albino mice exposed to the whole body gamma radiation. Indian J Nucl Med.

[9] Nguyen P.A. (2025). Quercetin and its derivatives from lotus (Nelumbo nucifera) seedpod extract combat radioresistance by suppressing ACSL4. Biofactors.

[10] Prades-Sagarra È. (2025). The radiosensitizing effect of Caffeic Acid Phenethyl Ester in breast cancer is dependent on p53 status. Radiother Oncol.

[11] Li C. (2020). Vitexin ameliorates chronic stress plub high fat diet-induced nonalcoholic fatty liver disease by inhibiting inflammation. Eur J Pharmacol.

[12] Li C. (2025). Exploring the Anticancer potential of MonoHER (7-Mono-O-(β-Hydroxyethyl)-Rutoside): Mitochondrial-Dependent Apoptosis in HepG2 Cells. Curr Issues Mol Biol.

[13] Vomund S. (2017). Nrf2, the master regulator of anti-oxidative responses. Int J Mol Sci.

[14] Jaiswal A.K. (2004). Nrf2 signaling in coordinated activation of antioxidant gene expression. Free Radic Biol Med.

[15] Banáth J.P., Macphail S.H., Olive P.L. (2004). Radiation sensitivity, H2AX phosphorylation, and kinetics of repair of DNA strand breaks in irradiated cervical cancer cell lines. Cancer Res.

[16] Ashraf S. (2024). Modulation of ATM enhances DNA repair in G2/M phase of cell cycle and averts senescence in Fuchs endothelial corneal dystrophy. Commun Biol.

[17] Feinendegen L.E. (2005). Evidence for beneficial low level radiation effects and radiation hormesis. Br J Radiol.

[18] Zhao Y. (2015). Ionizing radiation-induced adaptive response in fibroblasts under both monolayer and 3-dimensional conditions. PLoS One.

[19] Bugała E., Fornalski K.W. (2025). Radiation adaptive response for constant dose-rate irradiation in high background radiation areas. Radiat Environ Biophys.

[20] Yao Q. (2015). Late-responding normal tissue cells benefit from high-precision radiotherapy with prolonged fraction delivery times via enhanced autophagy. Sci Rep.

[21] Mortazavi S. (2013). Window theory in non-ionizing radiation-induced adaptive responses. Dose Response.

[22] Azzam E.I., Raaphorst G.P., Mitchel R.E. (1994). Radiation-induced adaptive response for protection against micronucleus formation and neoplastic transformation in C3H 10T1/2 mouse embryo cells. Radiat Res.

[23] Mothersill C., Seymour C. (2004). Radiation-induced bystander effects and adaptive responses–the Yin and Yang of low dose radiobiology?. Mutat Res.

[24] Iyer R., Lehnert B.E. (2002). Low dose, low-LET ionizing radiation-induced radioadaptation and associated early responses in unirradiated cells. Mutat Res.

[25] Feinendegen L.E. (2014). Evidence for beneficial low level radiation effects and radiation hormesis. Br J Radiol.

[26] Kanani A. (2025). Adaptive response: a scoping review of its implications in medicine, space exploration, and beyond. Dose-Response.

[27] Zhao X. (2017). Effects of low-dose radiation on adaptive response in colon cancer stem cells. Clin Transl Oncol.

[28] Wang S. (2013). Effect of low-dose X-ray radiation on adaptive response in gastric cancer cell. Chin-Ger J Clin Oncol.

[29] Jiang H. (2008). Low-dose radiation does not induce proliferation in tumor cells in vitro and in vivo. Radiat Res.

[30] Farhadi S. (2022). DNA double-strand break repair and adaptive responses of low-dose radiation in normal and tumor lung cell lines. Mut Res/Genetic Toxicol Environ Mutag.

[31] Kilemade M., Lemon J., Boreham D. (2008). Characteristics of the adaptive response in cultured salmon cells exposed to ionizing radiation. Environ Mol Mutagen.

[32] Wang X.-C. (2021). The adaptive responses in non-small cell lung cancer A549 cell lines induced by low-dose ionizing radiation and the variations of miRNA expression. Dose-Response.

[33] Yang W. (2009). TH-D-BRD-04: increased tumor radioresistance by imaging doses from volumetric image guided radiation therapy. Med Phys.

[34] Hyland W.B. (2014). Investigation into the radiobiological consequences of pre-treatment verification imaging with megavoltage X-rays in radiotherapy. Br J Radiol.

[35] Azzam E.I., de Toledo S.M., Little J.B. (2004). Stress signaling from irradiated to non-irradiated cells. Curr Cancer Drug Targets.

[36] Feinendegen L. (2005). Low doses of ionizing radiation: relationship between biological benefit and damage induction. a synopsis. World J Nucl Med.

[37] Chen M., Linstra R., van Vugt; M.A.T.M. (2022). Genomic instability, inflammatory signaling and response to cancer immunotherapy. Biochim. et Biophys. Acta (BBA) – Rev. Cancer.

[38] Mitchell S.A. (2004). Bystander effect and adaptive response in C3H 10T(1/2) cells. Int J Radiat Biol.

[39] Lee S.J. (2000). Adaptive response is differently induced depending on the sensitivity to radiation-induced cell death in mouse epidermal cells. Cell Biol Toxicol.

[40] Jomova K. (2023). Reactive oxygen species, toxicity, oxidative stress, and antioxidants: chronic diseases and aging. Arch Toxicol.

[41] Osburn W.O., Kensler T.W. (2008). Nrf2 signaling: an adaptive response pathway for protection against environmental toxic insults. Mutat Res.

[42] Kensler T.W., Wakabayashi N., Biswal S. (2007). Cell survival responses to environmental stresses via the Keap1-Nrf2-ARE pathway. Annu Rev Pharmacol Toxicol.

[43] Vomund S. (2017). Nrf2, the master regulator of anti-oxidative responses. Int J Mol Sci.

[44] Valdiglesias V. (2013). γH2AX as a marker of DNA double strand breaks and genomic instability in human population studies. Mut Res/Rev Mut Res.

[45] Podhorecka M., Skladanowski A., Bozko P. (2010. 2010.). H2AX Phosphorylation: its role in DNA damage response and cancer therapy. J Nucleic Acids.

[46] Meyn M.S. (1995). Ataxia-telangiectasia and cellular responses to DNA damage. Cancer Res.

[47] Burma S. (2001). ATM phosphorylates histone H2AX in response to DNA double-strand breaks. J Biol Chem.

[48] Stiff T. (2004). ATM and DNA-PK function redundantly to phosphorylate H2AX after exposure to ionizing radiation. Cancer Res.

[49] Yang G. (2016). Distinct biological effects of low-dose radiation on normal and cancerous human lung cells are mediated by ATM signaling. Oncotarget.

[50] Park S.H. (1999). Different induction of adaptive response to ionizing radiation in normal and neoplastic cells. Cell Biol Toxicol.

[51] Dai X. (2009). Low dose hyper-radiosensitivity in human lung cancer cell line A549 and its possible mechanisms. J Huazhong Univ Sci Technolog Med Sci.

[52] Shiloh Y., Ziv Y. (2013). The ATM protein kinase: regulating the cellular response to genotoxic stress, and more. Nat Rev Mol Cell Biol.

[53] Yang G. (2016). Distinct biological effects of low-dose radiation on normal and cancerous human lung cells are mediated by ATM signaling. Oncotarget.

[54] Maurya D.K., Devasagayam T.P.A. (2010). Antioxidant and prooxidant nature of hydroxycinnamic acid derivatives ferulic and caffeic acids. Food Chem Toxicol.

[55] Hosseinimehr S.J. (2010). Flavonoids and genomic instability induced by ionizing radiation. Drug Discov Today.

[56] Metodiewa D. (1999). Quercetin may act as a cytotoxic prooxidant after its metabolic activation to semiquinone and quinoidal product. Free Radic Biol Med.

[57] Mirza M.A. (2023). Quercetin as a therapeutic product: evaluation of its pharmacological action and clinical applications-a review. Pharmaceuticals (Basel).

[58] Salehi B. (2020). Therapeutic potential of quercetin: new insights and perspectives for human health. ACS Omega.

[59] Proshkina E., Shaposhnikov M., Moskalev A. (2020). Genome-protecting compounds as potential geroprotectors. Int J Mol Sci.

[60] Merlin J.P.J., Rajan S.S., Abrahamse H. (2025). Photodynamic therapy and dietary antioxidants: a dual strategy for genome stability and DNA damage repair. Cancer Med.

