# Supplementary material for: Differential regulation of radioadaptation by quercetin between human normal and cancer cells
Source: Clin Transl Radiat Oncol. 2025 Dec 17;57:101099. doi: 10.1016/j.ctro.2025.101099 (PMC12794430; doi:10.1016/j.ctro.2025.101099)
Supplement: Supplementary Data 1 [file mmc1.docx]

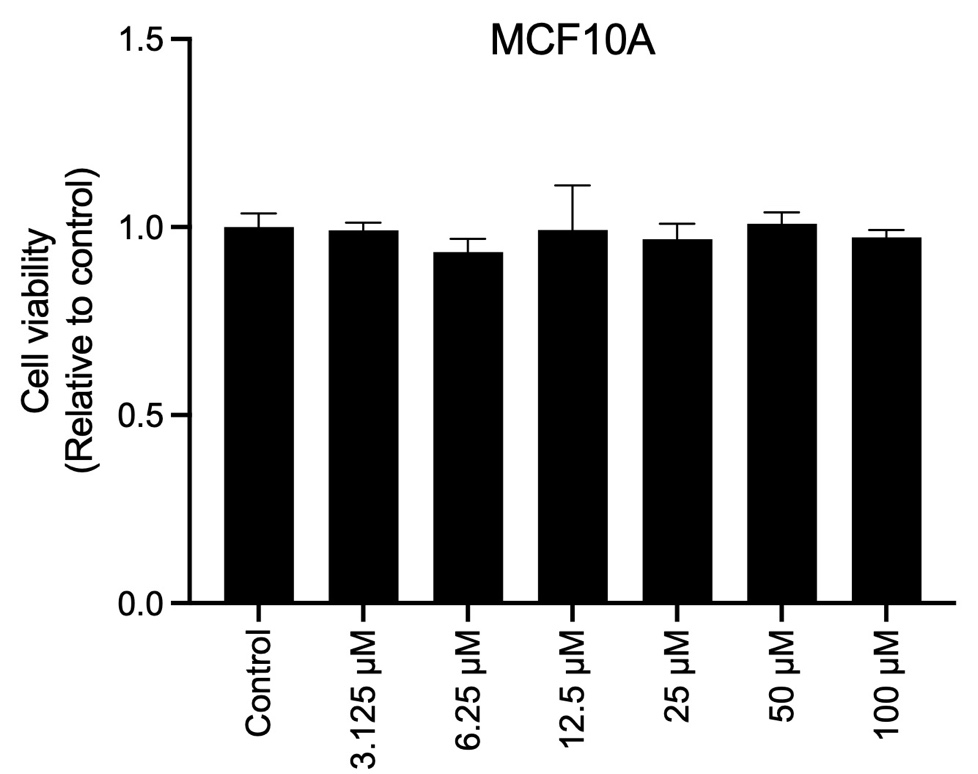


Supplementary figure 1. MCF10A cell viability following treatment with various concentrations of monoHER was assessed using the MTT assay.
